# A Rare Case of Ewing Sarcoma of the Maxilla and a Literature Review of Similar Cases

**DOI:** 10.7759/cureus.90342

**Published:** 2025-08-17

**Authors:** Abdul Moiz Khan, Rehan Ali, Saliq Naeem, Amna Ehtisham, Shah Rukh

**Affiliations:** 1 Internal Medicine, Sahiwal Medical College, Sahiwal, PAK; 2 General Medicine, Sahiwal Medical College, Sahiwal, PAK; 3 Internal Medicine, Allama Iqbal Medical College, Lahore, PAK

**Keywords:** anterior maxilla, ewing sarcoma (es), immunohistochemistry staining, pediatric bone tumor, small blue round cell tumor

## Abstract

Ewing sarcoma (ES) is a small, round, blue cell malignant neoplasm that rarely occurs in the craniofacial skeleton, with presentation in the maxilla being exceedingly rare. We report the case of a 14-year-old girl with a two-year history of slowly progressive facial swelling that has recently become greatly enlarged. Imaging revealed an aggressive expansile left maxillary lesion with soft-tissue involvement and internal calcifications. Surgical resection was performed, and histopathological analysis showed sheets of homogenous round cells with minimal cytoplasm and round nuclei. Immunohistochemical staining for CD99, CD56, and NKX2.2 was positive, while staining for epithelial, muscle, and neural markers was negative, thereby confirming the diagnosis of ES. This case highlights the diagnostic difficulty of ES in unusual sites such as the maxilla, where it can mimic odontogenic or fibro-osseous lesions. Early diagnosis with histopathology and immunohistochemistry is important for appropriate management, which usually includes surgical resection followed by chemotherapy.

## Introduction

Ewing sarcoma (ES) is a small, blue, round-cell tumor [1]. In 1921, James Ewing was the first to describe ES, referring to it as diffuse endothelioma of bone, as he believed the tumor originated from the vascular component of bone. Although the exact source of ES is still not fully understood, it is thought that the tumor may come from neuroectodermal cells. Some other possible sources that have been suggested include the early-stage reticular cells and the primitive mesenchymal cells found in the bone marrow [2]. Although uncommon in adults, classic bone ES is the second most common primary bone tumor found in children, following osteosarcoma in frequency [3].

ES usually develops in the long bones of the limbs, such as the femur or humerus, and accounts for approximately 4-6% of all primary bone tumors. ES is uncommon in locations outside the bones, and it is particularly rare in the head and neck area, accounting for only 1-4% of all cases [4]. Within the head and neck region, the mandible and calvaria are the most frequently affected sites, while the paranasal sinuses (PNS) are the least commonly involved locations [5].

ES is also defined by characteristic gene fusions that generate oncogenic chimeric transcription factors. The prototypical fusion is *EWSR1::FLI1*, resulting from t(11;22)(q24;q12), which occurs in the majority of classic ES cases and drives tumorigenesis by dysregulating transcriptional programs. Variants of *EWSR1* fused to other ETS family members (for example, ERG) are also recognized. A related group of "Ewing-like" or round-cell sarcomas, now recognized as separate entities, harbor CIC-rearrangements (most commonly CIC::DUX4) or *BCOR* genetic alterations (for example, *BCOR::CCNB3*); these tumors often differ in morphology, immunophenotype, and clinical behavior from canonical *EWSR1*-fusion ES.

Herein, we present a rare case of ES that originated in the maxillary sinus and highlights the importance of keeping ES as a differential while coming across head and neck tumors.

## Case presentation

A 14-year-old female presented with swelling on the left side of the face near the distal part of the nose, persisting for two years following trauma. The patient noted that the swelling had started to increase in size. On extraoral examination, the swelling measured approximately 4 × 3 cm on the left side of the face, extending just lateral to the ala of the nose. On palpation, the swelling was somewhat hard, with a normal temperature, and it was non-tender, non-fluctuant, non-mobile, and non-compressible, with no palpable pulsation. Intraorally, the swelling was exophytic, involving the maxillary bone and gingiva, extending from the right maxillary central incisor (11) to the second premolar (25), along with expansion into the buccal and palatal bone.

In considering the clinical differential diagnosis for a destructive mass in the head and neck region of a young patient, several entities were evaluated. Rhabdomyosarcoma typically presents in children or adolescents with a rapidly enlarging mass, often accompanied by local pain or functional impairment, and may demonstrate a more infiltrative pattern on imaging. Lymphomas, particularly non-Hodgkin types, can present as painless swellings with systemic "B" symptoms and are often associated with lymphadenopathy elsewhere. Nasopharyngeal carcinoma typically originates from the nasopharyngeal mucosa, presenting with early symptoms such as nasal obstruction, epistaxis, or conductive hearing loss, and is more common in older adolescents and adults. Olfactory neuroblastoma typically originates from the upper nasal cavity and may cause nasal obstruction, anosmia, or epistaxis, with a characteristic "dumbbell" appearance on imaging when it extends into the anterior cranial fossa. Poorly differentiated carcinomas in this region, although rare in younger patients, may mimic sarcomas clinically but are often accompanied by mucosal lesions on endoscopy. Careful integration of clinical presentation, radiologic features, and histopathology was essential in narrowing the diagnosis toward ES in this case.

**Figure 1:**
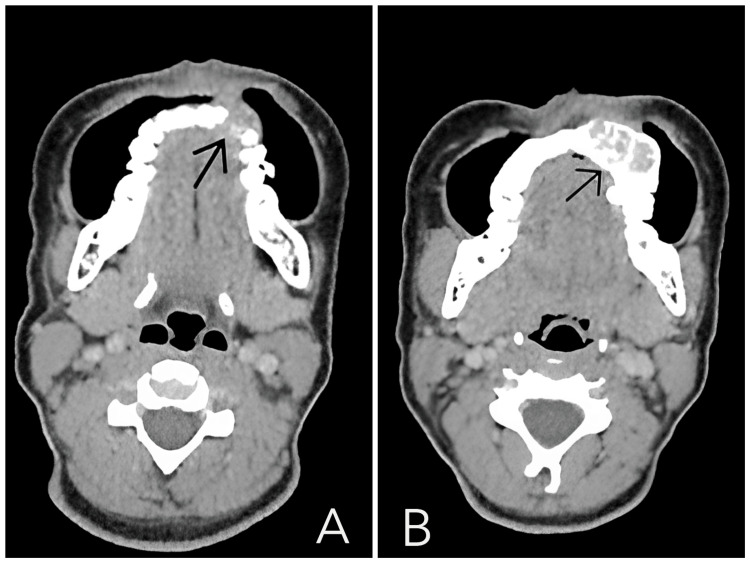
Axial CT of the face (soft-tissue window) demonstrating an expansile lytic maxillary mass (black arrows); biopsy-proven Ewing sarcoma in the (A) lower axial and (B) superior axial parts of the face. (A) Lower axial: Expansile, lytic lesion centered in the left upper alveolar arch just superior to the first and second premolars (black arrow). The mass measures approximately 28.8 × 20.4 × 24.2 mm (AP × transverse × craniocaudal) and closely abuts the premolar roots, with focal cortical thinning/erosion. (B) More superior axial: The lesion contains internal punctate/coarse calcifications (black arrow) and displaces the unerupted left maxillary canine superiorly. Appearance reflects an aggressive, mineralizing tumor with local bone destruction, and biopsy confirmed Ewing sarcoma.

A computed tomography (CT) scan revealed an aggressive-looking expansile lytic lesion with internal calcification in a left paramedian location within the left maxilla. It measured approximately 28.8 × 20.4 × 24.2 mm in AP × TR × CC dimensions. The lesion was centered on the left upper alveolar arch, just superior to the first and second premolars, abutting their roots (Figure 1).

**Figure 2:**
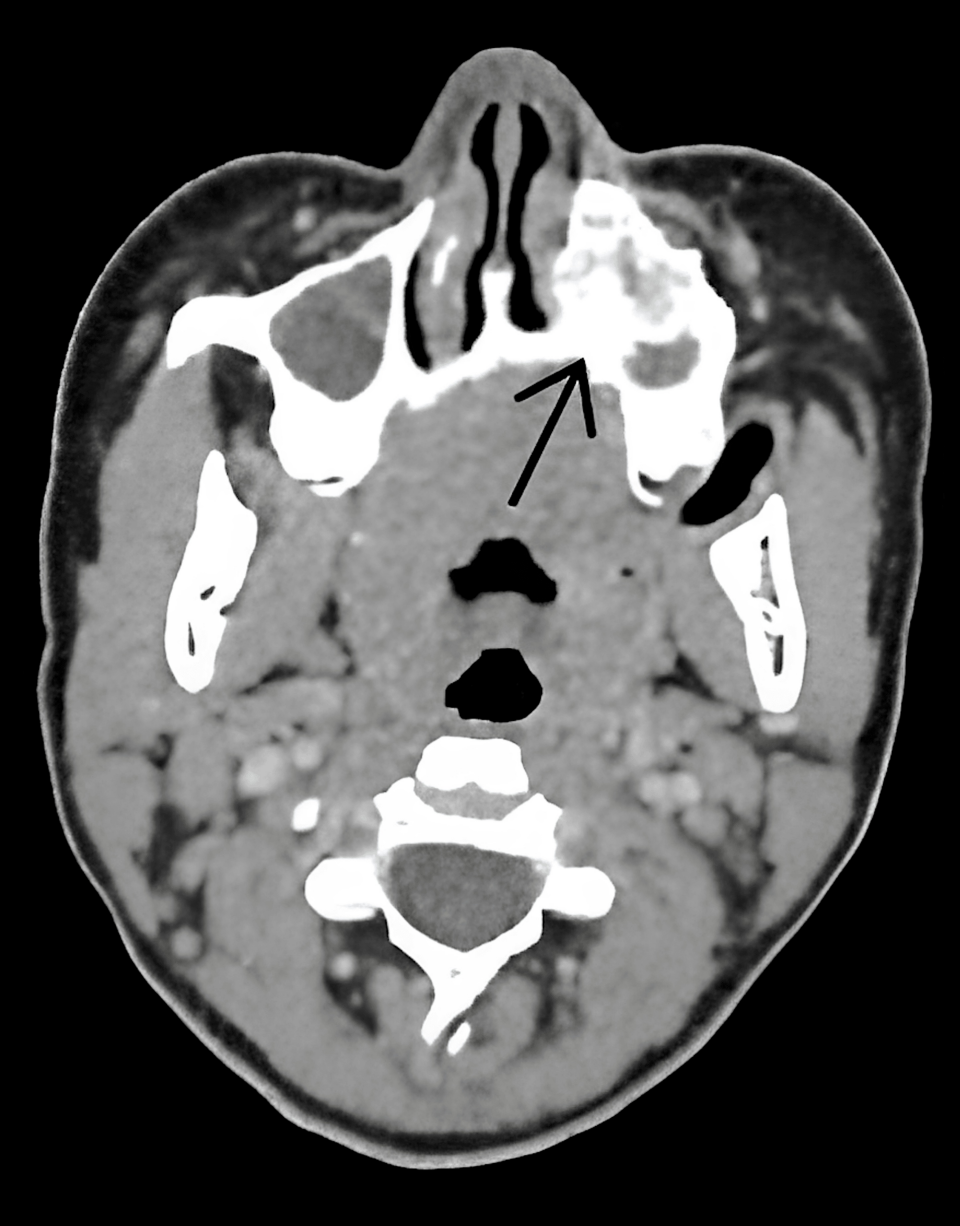
CT scan revealing no involvement of the left maxillary sinus by the tumor (black arrow). Coronal CT scan illustrating that the lesion spares the left maxillary sinus (black arrow). Although the floor of the maxilla is thinned, there is no extension into the sinus cavity. The lesion extends anteriorly into the overlying skin and subcutaneous tissues and inferiorly into the gingival mucosa.

Superiorly, it was in close approximation with the crown of an unerupted left canine tooth, which was displaced superiorly. Anteriorly, it was causing erosion of the cortex and extending into the overlying skin and subcutaneous plane, involving the soft tissue of the upper lip and cheek. Inferiorly, it extended into the gingival mucosa. Superiorly, it extended up to the floor of the maxilla, causing thinning, although no involvement of the left maxillary sinus was noted (Figure 2).

**Figure 3:**
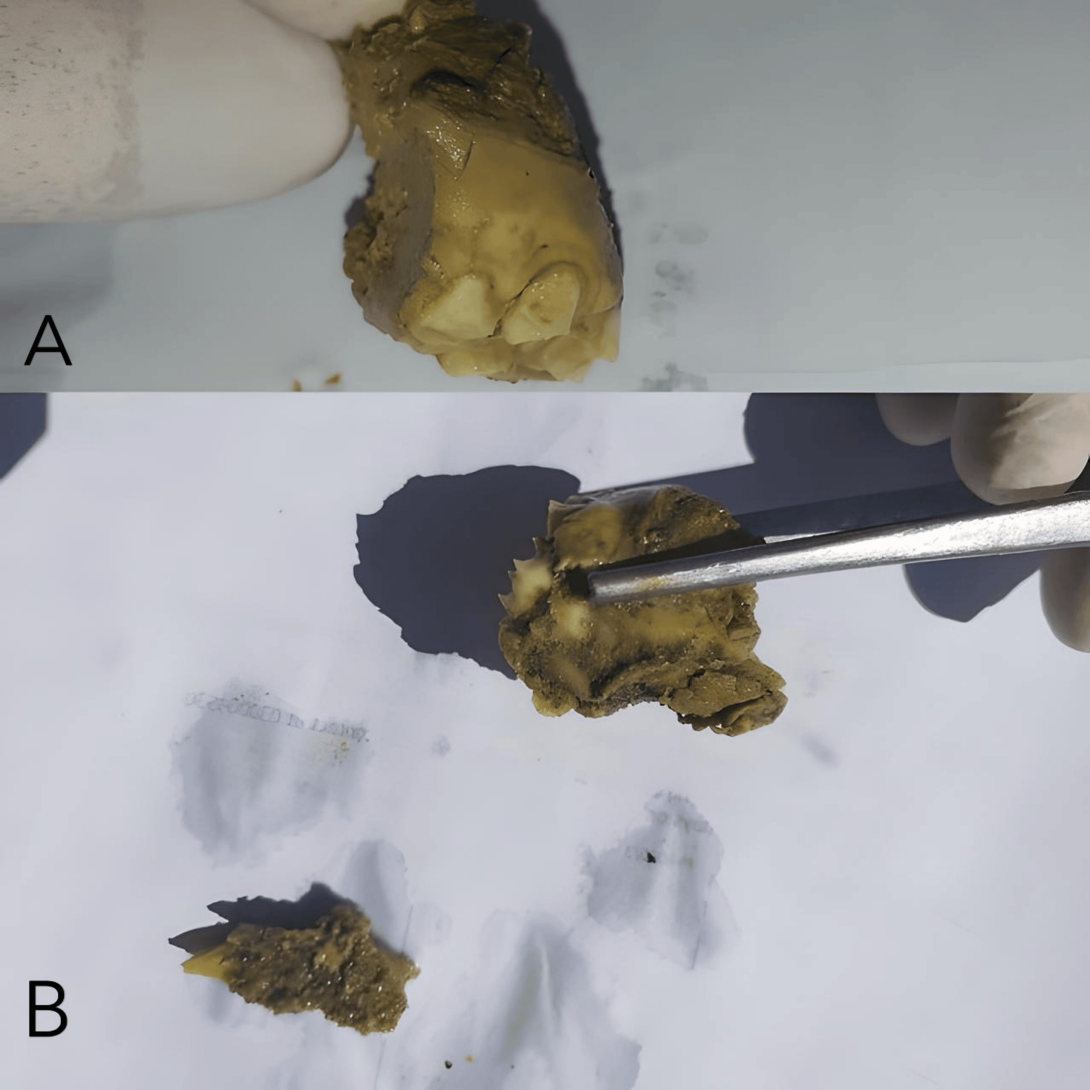
Gross resection specimen from the left maxilla (biopsy-proven Ewing sarcoma). (A) Close-up of tumor surface with adherent dentition; (B) specimen overview showing tooth-bearing fragments and tumor bulk. (A) Close-up view of the resected specimen showing an irregular, lobulated tan-brown soft-tissue mass with adherent necrotic/hemorrhagic material and multiple attached teeth (visible crowns and roots), consistent with a tumor arising from and destroying the alveolus. (B) Overview of the specimen fragments demonstrating portions of the mass with embedded/extracted teeth and surrounding soft tissue; the specimen measured 5 × 4 × 3 cm on gross inspection.

A surgical resection of the mass was planned. Written informed consent was obtained from the patient after the procedure and its risks and benefits were explained. The mass was resected from the maxilla, along with teeth 11, 12, 13, 21, 22, 23, 24, 25, and 26. Bone and soft tissue were resected up to the maxillary sinus, including resection of the sinus wall. The lesion was processed for histopathological evaluation. On gross examination, the specimen was irregular with attached soft tissue, measuring 5 × 4 × 3 cm (Figure 3).

**Figure 4:**
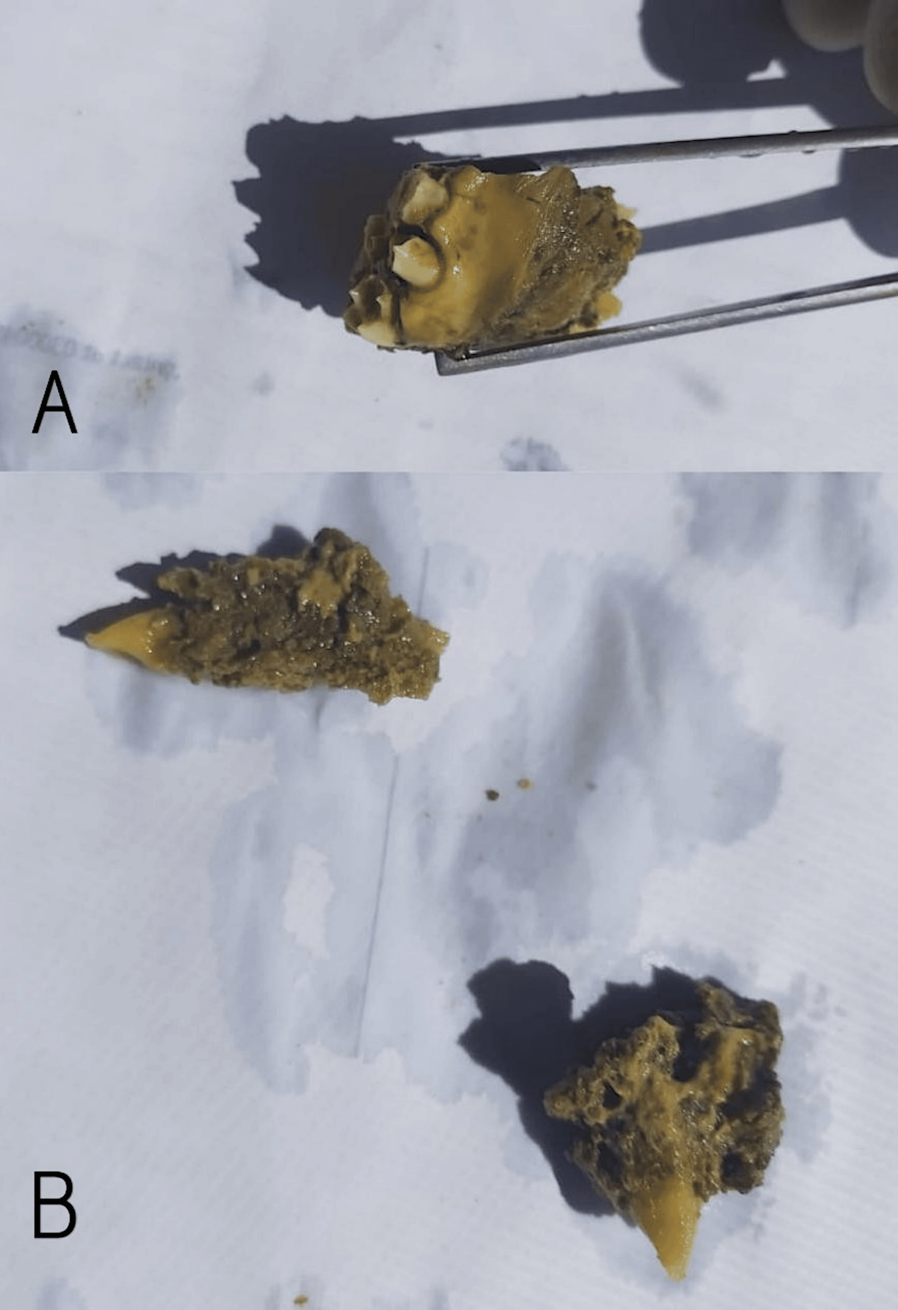
Cut sections of the resected left maxillary specimen (biopsy-proven Ewing sarcoma) demonstrating tumor infiltration of the alveolar bone with attached premolars and canines. (A) Close-up tooth-bearing fragment with tumor infiltration; (B) specimen fragments showing infiltrative cut surfaces and attached premolar/canine roots. (A) Close-up of a larger tooth-bearing fragment: the cut surface is soft, tan-brown, and irregular, with a tumor infiltrating around and into the tooth roots (premolars/canine visible). (B) Additional smaller fragments showing infiltrative tumor tracking along root surfaces and into adjacent bone; one fragment demonstrates the pointed canine apex projecting from the tumor mass.

Five to six teeth were present. Sectioning revealed the lesion had a soft consistency and extended into attached structures with bony involvement (Figure 4).

**Figure 5:**
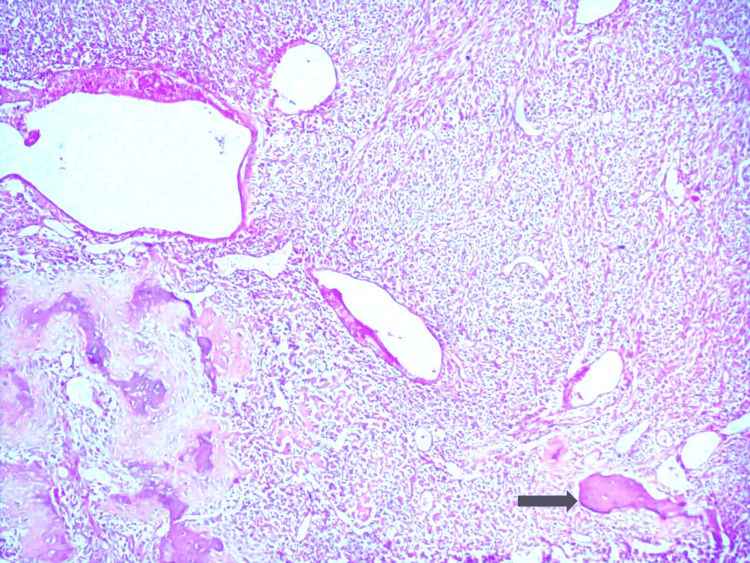
Malignant neoplasm showing round cells with scant cytoplasm and round nuclei with fragments of bone (black arrow). Hematoxylin and eosin (H&E)-stained section (original magnification ×40) showing sheets of uniform small round blue cells with scant cytoplasm and round nuclei, separated by thin fibrous septa. Fragments of bone are visible (black arrow), indicating osseous invasion. The nuclear chromatin is finely stippled, and nucleoli are inconspicuous, features characteristic of Ewing sarcoma.

On microscopic examination, the section showed infiltration by malignant neoplasm arranged in solid sheets separated by fibrous tissue. Individual cells were uniform and round to blue, with scant clear to eosinophilic cytoplasm, indistinct cytoplasmic membranes, round nuclei, finely stippled chromatin, and inconspicuous nucleoli. The tumor was seen invading the maxillary bony space. All soft tissue margins were free, and no lymphovascular invasion was seen (Figure 5).

**Figure 6:**
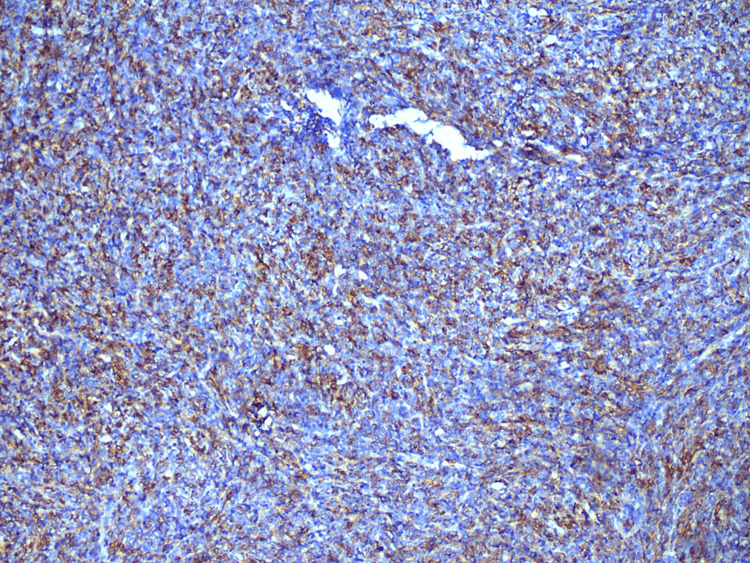
Cell showing positivity for CD99. Immunohistochemistry (IHC) for CD99 showing diffuse, strong membranous positivity in tumor cells. CD99 expression is a characteristic but not entirely specific finding for Ewing sarcoma, aiding in diagnosis when interpreted in conjunction with morphology and other markers.

**Figure 7:**
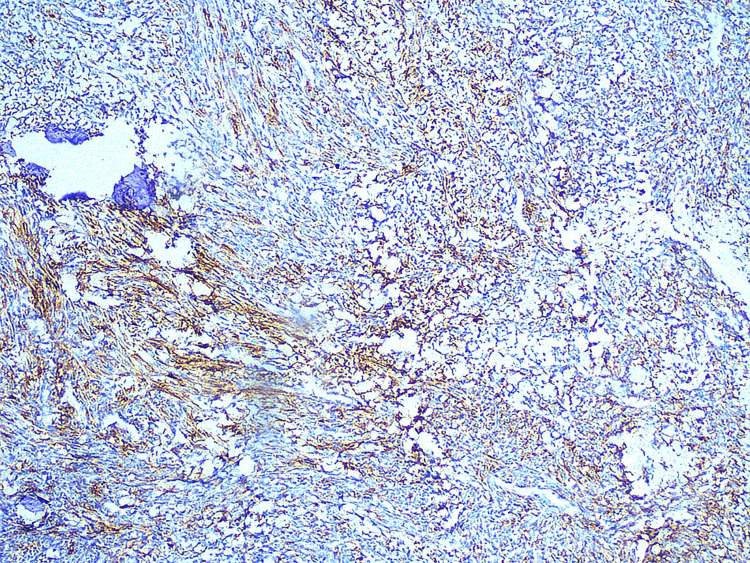
Cell showing positivity for CD56. IHC for CD56 revealing strong cytoplasmic positivity in tumor cells. CD56 is a neural cell adhesion molecule that is often expressed in Ewing sarcoma, supporting the neuroectodermal origin of the tumor.

**Figure 8:**
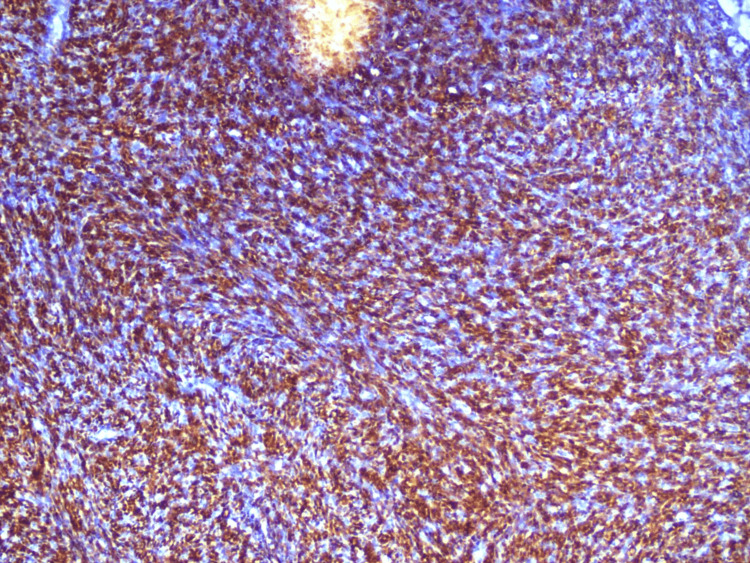
Cell showing positivity for NKX 2.2. IHC for NKX2.2 showing nuclear positivity in tumor cells. NKX2.2 is a transcription factor expressed in the majority of Ewing sarcoma cases and, in combination with CD99 and CD56, supports the diagnosis and helps exclude histologic mimics.

On immunohistochemistry, the tumor cells were positive for CD99 (Figure 6), CD56 (Figure 7), and NKX2.2 (Figure 8). They were negative for CK19, CD34, and NSE and Pan-CK as well as for Desmin and S100. Hence, a diagnostic confirmation of ES was made while ruling out other soft tissue tumors.

The patient underwent surgical excision of the tumor and had an uneventful postoperative recovery. She was given the vincristine, doxorubicin, and cyclophosphamide (VDC) regimen and was educated about the signs of recurrence, including new swelling, pain, or facial weakness, and was advised to perform regular self-examination. Follow-up imaging was not immediately warranted due to clear histology and margins, but was planned if clinical suspicion arose. Regular follow-ups were scheduled every six months. At the one-year review, she remained asymptomatic. The surgical scar was visible, and she reported complete symptom resolution.

The history of slow, indolent growth over a two-year period followed by recent rapid enlargement is atypical for ES, which more commonly presents with a short history of progressive symptoms lasting weeks to months. Such a prolonged initial course may reflect tumor location in a relatively silent anatomical space, thereby delaying symptom onset and diagnosis. During the recent period of rapid progression, the patient reported localized pain and swelling, accompanied by numbness in the adjacent facial region and mild functional limitations in mastication and speech. These details underscore the importance of considering ES even in cases with unusually protracted symptom evolution.

## Discussion

ES is a highly aggressive bone tumor with a documented resemblance to the primitive neuroectodermal tumor (PNET) in multiple aspects. ES predominantly affects the pediatric and adolescent population. Though it is the second most common primary malignant bone tumor in children, it is exceedingly rare in the head and neck region, comprising only 1-4% of all ES cases. Within this already rare distribution, involvement of the maxilla is even less frequent, making this case particularly notable [2].

A 14-year-old female presented with a persistent swelling on the left side of the face, initially noted after trauma and gradually enlarging over two years, with accelerated growth over the last six months. This clinical history highlights a common diagnostic challenge. ES in atypical locations often presents with non-specific symptoms and is easily mistaken for more common odontogenic or inflammatory conditions. Oftentimes, at the first medical consultation, the initial diagnostic suspicion is of a dental infection, a salivary gland tumor, or Burkitt's lymphoma [2]. This is reflected in the initial differential diagnoses, which included ameloblastoma, odontogenic myxoma, and mucoepidermoid carcinoma.

The radiologic findings of an aggressive, expansile lytic lesion with internal calcifications localized to the left maxilla further suggested a malignant process. Notably, the lesion extended into surrounding structures, including the soft tissue of the upper lip and cheek, gingival mucosa, and floor of the maxilla, without breaching into the maxillary sinus. These features are consistent with the destructive and infiltrative nature of ES.

Histologically, the tumor demonstrated uniform small round cells with scant cytoplasm, round nuclei, and finely stippled chromatin, hallmark features of ES. Immunohistochemical analysis with positive staining for CD99 was crucial for a definitive diagnosis of ES. Positive staining for CD56 and NKX2.2 and negative results for markers such as CK19, Pan-CK, Desmin, and S100 effectively ruled out other small round-cell tumors, including lymphomas, rhabdomyosarcoma, and neuroblastoma. Immunohistochemistry remains essential for screening: diffuse membranous CD99 expression is seen in most ES cases but is not entirely specific, while NKX2-2 is a useful adjunct that increases specificity when used together with CD99.

Definitive classification generally requires demonstration of a pathognomonic gene rearrangement by molecular methods, i.e., commonly FISH (EWSR1 break-apart), RT-PCR for specific fusion transcripts, or targeted next-generation sequencing panels that detect a broad range of sarcoma fusions. FISH is widely used as a sensitive screening tool on FFPE tissue, while RT-PCR/NGS can characterize the exact fusion partner when needed [6].

For ES in the head and neck region, the standard of care involves a multimodal therapeutic approach. After the initial diagnostic biopsy, a combination of surgical resection, systemic chemotherapy, and localized radiotherapy is employed. Such an integrated treatment strategy has been associated with improved long-term survival.

Nevertheless, the overall prognosis for ES remains guarded, primarily due to its propensity for early hematogenous dissemination, particularly to the lungs, which often occurs within a few months of diagnosis. Recent evidence highlights tumor burden at presentation as a significant prognostic indicator, with lower tumor volumes correlating with more favorable outcomes.

In summary, this case underscores the importance of including ES in the differential diagnosis of rapidly enlarging maxillofacial lesions in young patients, even in the absence of systemic symptoms. Increasing numbers of cases of ES in the orofacial region have been reported over the last 15 years, which supports the argument presented here. Raghani et al. [6] discussed the case of ES of the maxilla and zygoma in a 13-year-old male patient. Astekar et al. described the case of a 24-year-old male patient with a four-year-old facial swelling that progressed to epistaxis and was later confirmed to be ES of the maxilla [7]. A comprehensive literature review of the past five years revealed a few reported cases of ES of the face, highlighting its diverse clinical presentations, histopathological features, and outcomes [9-13]. All cases exhibited "small round blue cell" histology characteristic of ES and strong CD99 positivity. Imaging typically shows an aggressive sinus mass (often osteolytic) with an invasion of adjacent structures. Treatment was uniformly multimodal (chemotherapy ± surgery and/or radiation). Follow-up ranged 6-12 months (when reported), with most patients achieving remission; only the adult case with metastatic spread fared poorly. Key adverse prognostic factors include large tumor size and the presence of metastases at diagnosis. For example, the 46-year-old patient who developed bone metastases had a worse course. In contrast, smaller, localized tumors that are completely resected with negative margins and treated with intensive chemotherapy (and radiation when appropriate) often achieve long-term control. These cases are summarized in Table 1.

**Table 1:** Ewing sarcoma of the maxilla: reported cases (2019–2024). CBCT: cone-beam computed tomography; ES: Ewing sarcoma; PET/CT: positron emission tomography/computed tomography; VDC/IE: vincristine, doxorubicin, and cyclophosphamide/ifosfamide and etoposide; IOPA: intraoral periapical radiograph

Study	Age/Sex	Presentation (Symptoms)	Tumor Location & Size	Imaging Findings	Histopathology	Immunoprofile	Treatment	Follow-up	Outcome
Chin et al. [9]	46 M	1 month of right nasal obstruction, epistaxis, and right-eye tearing	Friable mass in the anterior right nasal cavity with involvement of the right maxillary sinus mucosa (no bony erosion)	Contrast-enhanced CT: soft-tissue mass at the floor of the right anterior nasal cavity and mucosal thickening in the right maxillary sinus; no bone destruction or distant metastases	Sheets of small, round blue cells (poorly differentiated round-cell tumor)	CD99 (+, strong membranous), FLI-1 (+)	Endoscopic medial maxillectomy with modified Denker's approach; six cycles of chemotherapy (VDC/IE)	Post-treatment: short-term monitoring (follow-up duration not specified)	Developed bone metastases on follow-up bone scan (poor prognosis)
Kewalramani et al. [10]	23 M	1.5 months of left facial swelling (maxillary region); no pain	Diffuse swelling of the left maxillary sinus, extending into the ethmoid and sphenoid sinuses (breach of the infraorbital margin)	CBCT: expansile soft-tissue mass in left maxillary sinus (involving lateral, medial, superior walls and ostium), extending into nasal cavity; PET/CT: hypermetabolic lesion localized to left maxilla	Neoplastic small round blue cells arranged in sheets (consistent with ES)	CD99 (membranous+), WT-1 (+), FLI-1 (+), ERG (+), focal CK (+), weak CD31 (+)	Multi-agent chemotherapy (alternating VDC/IE for six cycles); no surgery or radiation mentioned	9 months follow-up with regular imaging	Complete response: Post-chemo PET/CT showed resolution of the mass with no metastases; disease-free at 9 months
Sharma et al. [11]	13 M	1 week of right mid-face swelling and mild pain after the extraction of the upper right first molar	Ill-defined, expansile osteolytic lesion in the right posterior maxilla, extending into the right maxillary sinus; floating tooth appearance	IOPA radiograph: missing right #17, severe alveolar bone loss; CBCT: expansile osteolytic lesion into sinus; PET-CT (post-treatment): regression and no residual uptake	Malignant round-cell tumor infiltrating the submucosa; sheets of uniform small round blue cells with scant cytoplasm	Vimentin (+), CD99 (+), FLI-1 (+)	Chemotherapy (VDC/IE, six cycles) + radiotherapy (5 fractions) + adjuvant chemotherapy (5 cycles)	6 months of follow-up; asymptomatic on exam; CBCT shows trabecular bone formation	No recurrence at 6 months
Hamid et al. [12]	26 M	2+ years of left nasal bleeding, obstruction, rhinorrhea	Polypoid mass arising from the left maxillary sinus, extending into the nasal cavity and choana; no external swelling	Contrast CT: isolated left maxillary sinus mass, no regional or distant metastasis	Uniform small round blue cells (sheets), confirming ES	Vimentin (+), CD99 (+), FLI-1 (+); negative for CK, S-100, desmin	Neoadjuvant chemotherapy (VDC/IE, nine cycles) + endoscopic medial maxillectomy	12 months post-therapy surveillance	Complete remission: No evidence of disease at 12 months
Cherraqi et al. [13]	11 F	1 month of left cheek swelling with pain, nasal obstruction, rhinorrhea; slight weight loss	Large mixed lytic-sclerotic lesion in the left maxillary sinus, destroying sinus walls and alveolus, extending into the nasal cavity	CT: heterogeneous lesion with bone lysis (orbit floor, sinus walls, palate) and nasal cavity extension	Clusters of small, round blue cells with fine chromatin (consistent with ES)	CD99 (+, strong/diffuse)	Neoadjuvant chemotherapy (six cycles VDC/IE)—partial (50%) tumor regression, followed by VAC + radiotherapy (17 fractions)	Post-therapy CT: stable residual lesion; ongoing follow-up	Stable disease: No progression

These findings underscore that tumor size, margin status, and metastasis are critical prognostic indicators in maxillary ES. Early imaging and biopsy, followed by immunohistochemical confirmation, are critical steps for accurate diagnosis and timely intervention. Overall, aggressive multimodal therapy and clear surgical margins are emphasized for optimal outcomes.

Given the rarity of ES in the maxillary region, reporting such cases contributes significantly to the existing literature, aiding in the recognition, diagnosis, and timely management of this aggressive malignancy in unusual locations.

## Conclusions

Multimodal therapy (surgery and chemotherapy alongside radiotherapy) remains the optimal treatment for ES, ensuring ideal patient outcomes. This case emphasizes the need for thorough histopathological evaluation to distinguish ES from other head and neck neoplasms. While most cases are treatable by multimodal therapy, the potential for local recurrence necessitates long-term follow-up to monitor for any signs of recurrence. Although the patient remained recurrence-free at the one-year follow-up, extended surveillance is recommended according to the cases' risk profile, particularly for tumors with rare histological subtypes and locations, to ensure early detection of delayed recurrence or malignant transformation.
